# Associations between maternal fish intakes, maternal and cord PUFA and longitudinal measures of child weight at birth, 20 months and at 7 and 13 years of age

**DOI:** 10.1017/S0007114526106369

**Published:** 2026-02-05

**Authors:** James E. McMullan, Alison J. Yeates, Philip J. Allsopp, Maria S. Mulhern, Marie C. Conway, Toni Spence, J.J. Strain, Edwin van Wijngaarden, Gary Myers, Emelyn Shroff, Juliette Henderson, Conrad Shamlaye, Emeir M. McSorley

**Affiliations:** 1 Nutrition Innovation Centre for Food and Health (NICHE), Ulster University, UK; 2 School of Biological, Health and Sport Sciences, Technological University Dublin, Republic of Ireland; 3 School of Medicine and Dentistry, University of Rochester, Rochester, NY, USA; 4 The Ministry of Health, Mahe, Republic of Seychelles

**Keywords:** Fish consumption, PUFA, obesity, pregnancy

## Abstract

Prenatal exposure to PUFA has been associated with child weight at birth and may have a persistent effect on adiposity development across childhood. Fish is the richest dietary source of n-3 PUFA, albeit few studies have investigated associations between maternal fish consumption during pregnancy and child weight. This study examines associations between maternal fish consumption and prenatal PUFA status (*n*-3 and *n*-6), with longitudinal measures of child weight in the high fish-eating Seychelles Child Development Study Nutrition Cohort 2. Maternal fish consumption during pregnancy was assessed using a Fish Use Questionnaire administered at 28 weeks’ gestation. Serum PUFA were quantified in maternal blood collected at 28 weeks’ gestation and in cord blood collected at delivery. Birth weight was measured at delivery and classified according to WHO growth standards (*n* 1185). Child length/height (m) and weight (kg) were recorded at 20 months (*n* 1182), 7 (*n* 1167) and 13 (*n* 878) years. Child BMI was classified according to *z*-scores. Maternal total fish consumption (range: 0·0–584·71 g/d) was not associated with child weight at any age. At 7 and 13 years, maternal total *n*-6 PUFA were associated with increased risk of overweight/obesity (7 years; OR = 1·62, *p* = 0·037, 13 years; OR = 2·05, *p* = 0·005). Lower (<0·071 mg/ml) cord DHA concentrations were associated with a greater likelihood of being large for gestational age (LGA; >90th percentile) when compared with higher (>0·129 mg/ml) cord DHA concentrations (OR 4·17, *p* = 0·017). This study suggests that prenatal maternal *n*-3 and *n*-6 PUFA status may influence postnatal outcomes, including child adiposity from birth until adolescence.

Pregnancy is a period of rapidly increasing physiological demands, where optimal nutrition is crucial to support both maternal and fetal growth^(1)^. Substantial evidence suggests that the nutritional status of the mother and, therefore, the intra-uterine environment is a critical determinant of developmental plasticity, having significant implications for the development of chronic disease in later life^(2,3)^. Birth weight serves as an indicator of infant intra-uterine growth with epidemiological data demonstrating associations between abnormal birth weights and the development of several chronic diseases throughout the lifespan^(4–6)^. Postnatally, the increase in the number of adipocytes is highest during early life; however, adipose tissue accretion begins *in utero*, and thus pregnancy may be a sensitive period for influencing the trajectory of weight throughout childhood^(7,8)^.

Dietary components that have gained considerable attention for their roles in pregnancy and fetal development are the long-chain *n*-3 PUFA, EPA (20:5n-3) and DHA (22:6n-3). The *n*-3 PUFA are traditionally known for their anti-inflammatory properties and role in neurodevelopment. However, they have also been shown to be associated with greater birth weight, lower risk of pregnancy complications and a lower risk of preterm delivery^(9–11)^. Additionally, it has been proposed that the *n*-3 PUFA may have the ability to influence child weight throughout early life^(12,13)^. Evidence from animal models appears to suggest that prenatal exposure to arachidonic acid (AA; 20:4*n*-6), an *n*-6 PUFA, promotes adipogenesis via the production of biologically active lipid metabolites, also known as eicosanoids. These act in a pro-inflammatory manner stimulating the proliferation of adipocytes. *In utero* exposure to *n*-3 PUFA appears to counteract this effect through the production of more anti-adipogenic lipid mediators^(14,15)^.

Despite the postulated biological mechanism for the prenatal exposure of *n*-3 and *n*-6 PUFA in influencing child weight, data from human studies have shown conflicting results. Findings from the Growing Up in Singapore Towards healthy Outcomes (GUSTO) cohort showed positive associations between maternal plasma linoleic acid (LA; 18:2n-6) concentrations and birth weight and abdominal adipose tissue volume within the child^(16)^. In contrast, a Chinese birth cohort reported higher maternal erythrocyte AA and total *n*-6 PUFA to be associated with a lower risk of overweight and obesity at 2 years^(17)^. Studies investigating the *n*-3 PUFA have also yielded inconsistent findings, with some reporting negative associations with weight throughout childhood^(18)^ and others observing positive or null associations^(19,20)^.

During pregnancy, the developing fetus is entirely dependent on the maternal supply of PUFA, with circulating concentrations known to fluctuate throughout gestation^(21)^. Fish is one of the richest sources of *n*-3 PUFA within the diet and is known to contain several other nutrients essential for fetal growth, including vitamin D, Se and protein^(22)^. Generally, regulatory agencies advise the consumption of 2–3 portions of fish (4 oz) per week during pregnancy on the basis that fish high in methylmercury, an environmental neurotoxicant, are avoided^(23,24)^. Studies examining the associations between maternal fish consumption and birth weight remain inconsistent with some reporting favourable fetal growth amongst women who consume seafood during pregnancy, whilst others have reported negative or null associations^(25,26)^. Notably, fewer studies have assessed the associations between maternal fish consumption during pregnancy and offspring weight throughout childhood. Van den Berg *et al.* (2016) analysed mother–child data from the Dutch Prevention and Incidence of Asthma and Mite Allergy (PIAMA) cohort and found no evidence of an association between maternal fish consumption and BMI throughout childhood^(27)^; however, this was a cohort of relatively low fish consumers, and further research in large population-based cohorts with a wide range in maternal fish consumption is warranted.

Given the ambiguity surrounding the roles of maternal PUFA status on child weight and the paucity of data on the effects of maternal fish consumption, the primary aims of this study were to examine the associations between maternal fish consumption and prenatal PUFA status (*n*-3 and *n*-6), including maternal and umbilical cord blood concentrations, with child weight at birth and throughout childhood (20 months, 7 and 13 years) in the high fish-eating Seychelles Child Development Study (SCDS) Nutrition Cohort 2 (NC2). It was hypothesised that higher intakes of fish, and thus higher concentrations of the *n*-3 PUFA, would be associated with a lower risk of overweight/obesity throughout childhood, whilst higher prenatal exposures to the *n*-6 PUFA would be associated with increased child adiposity.

## Methods

### Study population

The SCDS is an ongoing multicohort observational study with the overall aim of investigating the risks and benefits of prenatal fish consumption on child neurodevelopment. Mothers were enrolled onto NC2 during their first antenatal visit (from 14 weeks of gestation) between 2008 and 2011. Recruitment took place at eight health centres on Mahè, the main island of the Republic of Seychelles. Inclusion criteria included being native Seychellois, ≥ 16 years of age, having a singleton pregnancy and having no obvious health concerns^(28)^.

### Ethical approval

This study was conducted according to the guidelines laid down in the Declaration of Helsinki, and all procedures involving human participants were approved by the Seychelles Ethics Board and the Research Subjects Review Board at the University of Rochester. Written informed consent was obtained from all participants.

### Fish consumption

Habitual fish consumption throughout pregnancy was assessed retrospectively through a Fish Use Questionnaire (FUQ) administered at 28 weeks’ gestation. The FUQ was designed specifically for the Seychellois population and focused on the fish species available in the Seychelles. Participants were asked to report the number of times per week during pregnancy they consumed specific types of fish with frequency of consumption reported as meals per week. The reported weekly consumption of fishmeals was then converted to grams per d using the average portion size for each fish species and multiplying it by the frequency of consumption per week and dividing by 7 to estimate daily intake of fish in g/d^(29)^. Fish consumption data were further categorised into oily fish, lean fish and total fish intakes^(30)^. The FUQ was also completed at the 7- and 13-year time points to assess habitual fish consumption throughout childhood and adolescence. The FUQ has been compared against a 4-d semi-quantitative food diary using Bland–Altman plots and cross-validation to compare mean differences in fish consumption reported from each assessment tool. This analysis showed good concurrence between the questionnaire and diary with most data well within the 95 % limits of agreement, which indicates that the FUQ is a valid tool for measuring fish consumption in this population (data unpublished).

### Blood sampling and PUFA analysis

Maternal non-fasting blood samples were collected at 28 weeks’ gestation, and cord blood samples were taken from the umbilical cord vein at delivery, from which serum was obtained. Maternal and cord samples were shipped at −80 °C to Ulster University for serum total PUFA analysis according to an adaptation of the Folch *et al*.^(31)^ method as previously described^(28)^. Briefly, fatty acid methyl esters were quantified using GC tandem MS (7890A–5975C; Agilent), with reference to heptadecanoic acid (C17:0) as an internal standard. Individual PUFA measured within the analysis included LA (18:2n-6), α-linolenic acid (ALA; 18:3n-3), AA (20:4n-6), EPA (20:5n-3) and DHA (22:6n-3) with results presented as milligrams per millilitre. ALA, EPA and DHA were summed to calculate the total *n*-3 PUFA, and total *n*-6 PUFA were calculated by the addition of LA and AA concentrations.

### Child weight

Birth weight (kilograms) was assessed at delivery by trained nurses and recorded to the nearest 2 decimal places using routine clinical procedures and standardised scales. Percentiles of birth weight according to gestational age were calculated based on the WHO Child Growth Standards^(32)^. Appropriate fetal growth for gestational age (AGA) was defined as a birth weight between the 10th and 90th percentiles. Children born with a birth weight <10th percentile for gestational age were classified as small for gestational age (SGA) and those born with a birth weight >90th percentile were deemed large for gestational age (LGA).

Child length/height (metres) and weight (kg) measurements were recorded by trained nurses at the 20-month, 7-year and 13-year follow-ups. Child *z*-scores were calculated using the WHO AnthroPlus software, and children were classified according to BMI *z*-scores. Children with a BMI *z*-score < −2·0 were classified as underweight and those with a BMI *z*-score >1·1 were deemed as overweight/obese. Normal weight throughout childhood was defined as a BMI *z*-score between −2·1 and 1·0^(33)^. All anthropometric measurements were recorded using standardised procedures and equipment calibrated by the Seychelles Bureau of Standards prior to and throughout the duration of the study.

### Covariates

Covariates were selected *a priori* based on the current literature and included maternal age, maternal BMI, socio-economic status, gestational age, child sex, parity (number of children) and alcohol use during pregnancy^(19,34–36)^. Child fish intakes were also considered as a covariate at 7 and 13 years. Owing to the low prevalence of smoking within the cohort (*n* 9), this was not adjusted for within the analysis. A questionnaire administered by trained nurses upon enrolment to the study was used to collect information on maternal age, parity and alcohol use throughout pregnancy (yes/no). Gestational age (weeks) and child sex were recorded at birth. At the 20-month follow-up, maternal socio-economic status was measured using the Hollingshead Social Status Index, modified for use within the Republic of Seychelles^(37)^. Data on prenatal BMI were unavailable within this cohort, and therefore, based on findings from a previous cohort that found a high correlation between early pregnancy BMI and postnatal BMI^(38)^; postnatal BMI was used as a surrogate for pre-pregnancy BMI. At the infants’ 20-month examination, maternal height and weight were recorded from which postnatal BMI was calculated (BMI = weight (kg)/height (m)^2^).

### Statistical analysis

**Figure 1.**
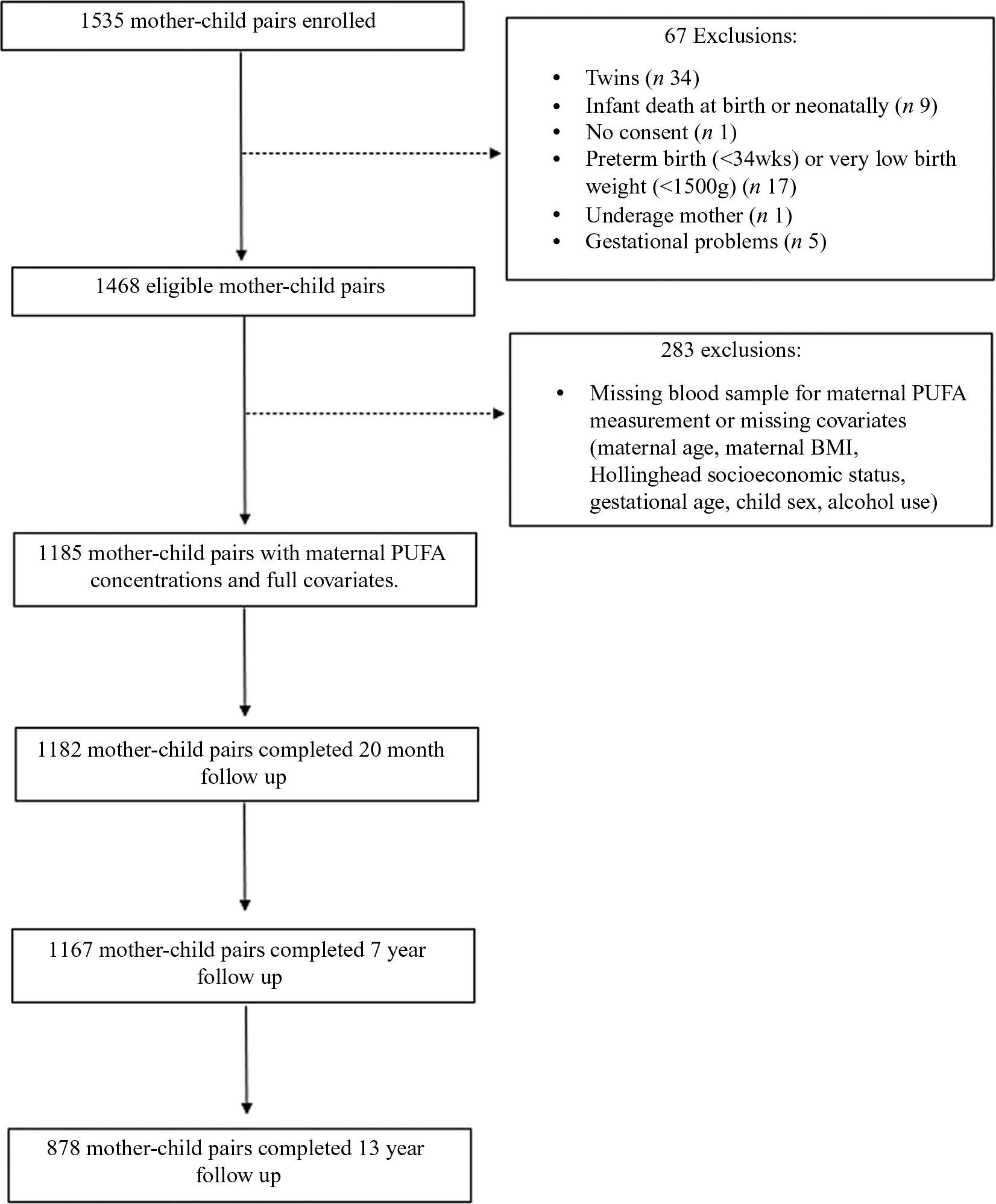
Description of exclusions and missing data for the current analysis of the Seychelles Child Development Study Nutrition Cohort 2.

A total of 1535 mothers were initially recruited onto the NC2 study. Mothers with extreme gestational age (preterm birth defined as <34 weeks gestation) and children with very low birth weight (<1500 g) were excluded from the current analysis (*n* 17). A total of 1185 mothers had PUFA concentrations measured at 28 weeks’ gestation and had available data on all covariates (Figure 1). Of the 1185 mothers included within the current analysis, 1124 had complete data on maternal fish intakes and 932 had complete PUFA concentrations measured within cord blood.

Statistical analysis was conducted using the Statistical Package for Social Sciences (SPSS, version 29.0, IBM). Data for all variables were tested for normality. Descriptive statistics were used to summarise the distribution of maternal and child characteristics. Associations between maternal fish consumption and maternal and cord PUFA concentrations and birth weight defined as SGA, AGA and LGA were assessed using multinominal regression analysis. Owing to the small number of underweight children with this cohort, BMI classifications throughout childhood were dichotomised (BMI *z*-score <2·0 or BMI *z*-score >2·0), and associations between the primary exposures and overweight/obesity (BMI *z*-score >2·0) at 20 months, 7 years and 13 years were examined using binary logistic regression models. Separate models were fitted using maternal fish consumption and maternal and cord PUFA concentrations as categorical variables with exposures grouped into quartiles based on sample distribution to aid in interpretability. All models were adjusted for maternal age, maternal BMI, gestational age, socio-economic status, child sex, alcohol use during pregnancy and parity. Models at 20 months, 7 years and 13 years were additionally adjusted for child fish intake at that time point. A *p*-value <0·05 was considered statistically significant throughout.

## Results

Table 1 displays the descriptive characteristics of the 1185 mother–child dyads included within the current analysis. Mothers had a mean age of 27·1 ± 6·3 years and were consuming on average 118·0 ± 78·8 g/d of fish throughout pregnancy (range: 0·0–584·7 g/d). Children comprised of 617 males and 568 females and had an average birth weight of 3·19 ± 0·46 kg. There were 209 (17·6 %) children classed as SGA, and only 38 (3·2 %) children were considered LGA. The mean birth weight of those classed as SGA and LGA was 2·62 ± 0·39 kg and 4·08 ± 0·42 kg, respectively. By 13 years, 305 (25·7 %) children were considered as overweight/obese. Maternal and cord PUFA concentrations are shown in Supplementary Table 1.

### Maternal fish consumption

Covariate-adjusted associations between maternal fish intakes and birth weight are presented in Table 2. There were no significant associations between maternal total fish, oily fish or lean fish intakes and SGA or LGA within the child.

There were also no significant associations between maternal total or oily fish intakes and child weight at 20 months, 7 years or 13 years (Table 3). There was, however, a significantly higher risk of overweight/obesity at 13 years amongst those consuming >57·14 g/d lean fish when compared with those in the lowest quartile of lean fish consumption (0·00 g/d) (OR 1·69, 95 % CI 1·09–2·62, *p* = 0·020), albeit this did not follow a dose–response trend with the second and third quartiles of lean fish consumption showing no significant association with overweight/obesity at 13 years.

### Maternal PUFA

Multinomial logistic regression analyses for covariate-adjusted associations between maternal PUFA concentrations and birth weight are presented in Supplementary Table 2. Only maternal AA concentrations in the lowest quartile (<0·148 mg/ml) were associated with a significantly lower risk of LGA (OR 0·26, 95 % CI 0·07–0·94, *p* = 0·041) when compared with the highest quartile (>0·261 mg/ml). No significant associations were found between any of the other maternal PUFA and LGA. No significant associations were noted between maternal concentrations of *n*-3 or *n*-6 PUFA and SGA.

Table 4 displays associations between maternal PUFA status and overweight/obesity at 20 months, 7 years and 13 years. At 20 months, children in the highest quartile of maternal LA concentrations (>1·050 mg/ml) had a significantly higher risk of childhood overweight/obesity when compared with those in the lowest quartile of maternal LA concentrations (<0·723 mg/ml) (OR: 1·45, 95 % CI 1·03–2·03, *p* = 0·032). Similar associations were found at 7 and 13 years, with the highest quartile of maternal LA and maternal total *n*-6 PUFA being associated with the highest risk of childhood overweight/obesity. Although there were no significant associations between any of the maternal *n*-3 PUFA and being overweight/obese throughout childhood, a higher maternal *n*6:*n*3 ratio (>5·011) was associated with a higher risk of being overweight/obese at both 20 months (OR: 1·67, 95 % CI 1·19–2·35, *p* = 0·003) and 7 years (OR: 1·75, 95 % CI 1·07–2·85, *p* = 0·025). There were no significant associations between the maternal *n*6:*n*3 ratio and being overweight/obese at 13 years.

#### Cord PUFA

Table 5 outlines associations between cord PUFA concentrations and childbirth weight. When compared with the highest cord DHA quartile (>0·129 mg/ml), only cord DHA concentrations <0·071 mg/ml were found to be associated with a significantly higher risk of LGA (OR 4·17, 95 % CI 1·30–13·44, *p* = 0·017). No other significant associations between any of the cord *n*-6 PUFA or the cord *n*6:*n*3 ratio and LGA were found. No significant associations were observed between any of the cord PUFA and SGA, albeit the third quartile of the cord *n*6:*n*3 ratio was associated with a lower risk of SGA (OR 0·54, 95 % CI 0·31–0·93, *p* = 0·027) when compared with the highest quartile; however, this may be considered a spurious finding given the lack of any dose–response.

Associations between cord PUFA and child weight at 20 months, 7 years and 13 years are displayed in Supplementary Table 3. There were no significant associations between any of the cord *n*-3 or *n*-6 PUFA and overweight/obesity throughout childhood.

## Discussion

In this high fish-eating cohort from the SCDS, total fish consumption throughout pregnancy was not associated with repeated measures of child weight at birth, 20 months, 7 years or 13 years. There were, however, several associations between the *n*-3 and *n*-6 PUFA and child weight. The lowest quartile of maternal AA concentrations (<0·148 mg/ml) was associated with a lower risk of LGA. Furthermore, higher maternal total *n*-6 PUFA concentrations were associated with an increased risk of overweight/obesity at 7 years and 13 years. In cord blood, DHA concentrations <0·071 mg/ml were associated with a significantly higher risk of LGA within the child. None of the observed associations between the cord *n*-3 or *n*-6 PUFA and child weight were evident at 7 or 13 years of age. Overall, these findings appear to suggest differing influences of lower cord *n*-3 and higher maternal *n*-6 PUFA status on child adiposity at birth until adolescence.

In the current study, there was no clear evidence of any associations between maternal total fish consumption and child weight at birth or throughout childhood, despite the uniquely high fish intakes within this cohort. Observational studies investigating the associations between maternal fish consumption and measures of child growth are few and have been overall inconclusive. Findings from this study appear to be consistent with a number of other mother–child cohorts, albeit lower fish consumers, where no associations were found^(27,39,40)^. In contrast, one larger Norwegian Mother and Child Cohort Study with a much lower average fish intake of 36 g per d reported positive associations between maternal seafood consumption and birth weight^(41)^. Furthermore, one pooled analysis of fifteen European and USA birth cohorts reported higher fish intakes (>3 times/week) to be associated with an increased risk of rapid infant growth and child overweight/obesity at 4 and 6 years which may be owing to contaminants found within fish^(36)^. We found that the consumption of >57·14 g/d lean fish was associated with an increased risk of overweight/obesity at 13 years; however, this association was not observed at any other time point, nor was it dose-responsive, and thus may be a chance finding. Studies which have reported a positive association between maternal fish intakes and child weight have noted that these associations appear to be dependent on the type of fish, with nutrients and contaminants within fish varying depending on the species and source which may also account for some variation between populations in addition to the differences in consumption patterns^(36,42)^. Nevertheless, fish is an important source of protein and nutrients required for growth and development both in pregnancy and childhood^(22)^, and uncertainty surrounding the benefits and/or risks of fish consumption during pregnancy on child body composition may lead to transitions towards more westernized-style diets which have been associated with metabolic complications including CVD^(43)^. This transition is particularly relevant amongst the Global South, with developing nations such as the Seychelles undergoing a period of rapid dietary transition resulting in lower fish intakes^(44)^. Given the limited and inconsistent data, alongside the public health implications, future longitudinal analyses using repeat measurements throughout childhood and which utilise direct measures of body composition are warranted to elucidate the relationships between maternal fish consumption during pregnancy and child adiposity.

Previous research has emphasised the importance of *n*-3 PUFA, primarily obtained in the diet from fish consumption, during pregnancy in relation to fetal growth, brain structure and neurodevelopment^(45,46)^. There was no evidence of any associations between the maternal *n*-3 PUFA and child weight at birth, 20 months, 7 years or 13 years, which confirms results from our previous analysis from the SCDS, where we did not detect any associations between maternal *n*-3 PUFA and birth outcomes^(34,35)^. The existing literature with regard to the associations between maternal *n*-3 PUFA status and child adiposity remains controversial and has primarily focused on early childhood which makes comparison to the current study challenging. Findings from the much larger Generation R Study noted higher maternal total *n*-3 PUFA concentrations within the second trimester were associated with a lower childhood body fat percentage and a lower android:gynoid fat mass ratio at 6 years^(47)^. Others have reported no associations between maternal *n*-3 PUFA concentrations and child fat mass at 6 years^(48)^. It is noteworthy that the two latter studies measured PUFA concentrations in plasma phospholipids, whereas in the current study maternal PUFA were measured in serum which makes comparisons more difficult. Moreover, 13 years may be a period of rapid pubertal growth where changes in the hormonal milieu result in adiposity accretion^(49)^. In this cohort, maternal fish consumption was not correlated with maternal PUFA status, which is also likely reflective of other factors influencing biomarker status. Aside from the differences in *n*-3 PUFA intakes, genetic variations can also influence PUFA metabolism and may also explain some of the inconsistencies across studies^(50)^. Future studies should consider the various genotypes which regulate PUFA metabolism when investigating the associations between maternal *n*-3 PUFA and child adiposity.

Within this cohort, maternal AA concentrations <0·148 mg/ml were associated with a lower risk of LGA at birth when compared with higher maternal AA concentrations. Additionally, the maternal *n*-6 PUFA were consistently associated with an increased risk of overweight/obesity throughout childhood. The current results are broadly consistent with the limited previous human studies^(47,48,51)^. The Southampton Women’s Survey reported positive associations between maternal plasma *n*-6 PUFA concentrations and child fat mass at 4 and 6 years^(48)^. Similar findings were also reported in the Generation R Study where higher maternal plasma *n*-6 PUFA were found to be associated with a higher childhood total body fat percentage and abdominal preperitoneal fat mass area^(47)^. Taken together, these findings, along with the current study, appear to suggest that higher prenatal exposures to the *n*-6 PUFA may influence adipogenesis development *in utero* and may be associated with an increase in child adiposity until early adolescence.

There are several existing mechanisms which may help to explain these observations. The *n*-6 PUFA are known to elicit a more pro-inflammatory immune response, leading to the production of pro-inflammatory lipid mediators known as eicosanoids^(52)^. These eicosanoids are highly biologically active, exerting effects on many cell types via cell membrane G-protein-coupled receptors^(53)^. For example, the *n*-6 PUFA are known to act as a precursor for prostacyclin (PG I_2_) which has been shown to bind to PPARγ which regulates adipogenesis via the proliferation of preadipocytes into mature adipocytes^(54,55)^. Moreover, there is also some evidence from murine models which suggests differing effects of fatty acids in the metabolic programming of the fetus *in utero* through epigenetic modification such as DNA methylation. Such epigenetic changes may influence growth in later life by influencing appetite control and energy balance^(56)^ and, thereby, may offer some plausible biological basis as to why associations were not apparent until later in childhood, albeit further work is needed to confirm this. We also noted an increased risk of overweight/obesity at 20 months and 7 years with a higher maternal *n*6: *n*3 ratio, which is generally associated with a greater pro-inflammatory immune response. The observed associations between the *n*-6 PUFA and child adiposity during childhood likely reflect the influence of the *n*-6 PUFA on the inflammatory milieu throughout pregnancy, and future work may need to measure eicosanoid production to improve mechanistic understanding in this area.

Typically, LA is abundant within the diet and found within several sources, including vegetable oils, nuts and seeds^(57)^. Endogenously, LA can undergo the elongation and desaturation pathway to the more biologically active *n*-6 PUFA, although AA is also found in high amounts in red meat from grain-fed animals^(58)^. Westernised-style diets are high in processed foods, red meat and eggs, which are typically higher in *n*-6 PUFA^(59)^. Previous research from our group has shown that this style of dietary pattern is most typically followed within this cohort of pregnant Seychellois women, reflecting the transition from the traditional Seychellois diet to a more westernized-style diet within the Republic of Seychelles^(30)^. The westernised dietary pattern has also been shown to be associated with an enhanced pro-inflammatory immune response and may be involved in promoting intracellular inflammatory pathways in adipocytes^(60)^. These findings have significant public health implications in light of the transition towards westernized-style diets throughout pregnancy and thus higher intakes of n-6 PUFA, particularly within the Global South. Such dietary changes may have important consequences for adiposity accretion and subsequently obesity development in the child in later life.

Although maternal *n*-3 PUFA were not found to be associated with child weight, cord DHA concentrations <0·071 mg/ml were associated with an increased risk of LGA. Much of the existing evidence has focused on maternal *n*-3 PUFA exposure earlier in pregnancy and the risk of preterm birth and gestational duration^(61)^, whereas fewer have examined cord PUFA status at delivery and its potential influence on fetal growth at term. One study of 847 mother–child pairs reported a negative association between cord erythrocyte DHA concentrations and birth weight^(62)^, whilst another, smaller observational study of 253 mother–child pairs also found a negative association between cord DHA concentrations and birth weight^(63)^. Whilst preterm births were excluded in the current analyses, and therefore the results may not be generalisable to pregnancies ending preterm or to outcomes related to gestational duration, the findings of the current study suggest that later-stage *n*-3 PUFA exposure may be important in relation to birth size. The *n*-3 PUFA are essential in mediating the immune response, acting as precursors for eicosanoids and docosanoids which are considered to be more anti-inflammatory^(64)^. The *n*-3 PUFA inhibit nuclear transcription factor kappa B, promoting a more anti-inflammatory immune response, which in turn may modulate adipokine secretion and thus lower adipogenesis and promote a more metabolically healthy phenotype^(65)^. Within this cohort, only thirty-eight (3·2 %) of children were considered LGA which is considerably lower than many other birth cohorts within Africa^(66,67)^ but is reflective of the Seychellois population which has an incidence of 4·4 %. The traditional Seychellois diet is rich in oily fish and fish dishes, which has been previously shown to be associated with a higher *n*-3 PUFA status^(30)^. Given both the rising incidence of LGA babies in many populations and the long-term metabolic complications that are often associated with LGA^(68)^, the unique dietary patterns within the Seychelles paired with the low incidence of LGA within this population may provide promising evidence of potential dietary interventions in helping to lower the risk of LGA within the child through increasing *n*-3 PUFA intakes throughout pregnancy.

It is important to recognise the differing associations between the maternal and cord PUFA observed within this study. For example, in this cohort, maternal *n*-6 PUFA were consistently associated with an increased risk of overweight/obesity at 7 and 13 years, whilst few associations were found with the cord PUFA. These differing associations are interesting, considering the developing fetus relies entirely on the maternal supply of PUFA owing to insufficient desaturase activity within the placenta or fetus to synthesise PUFA^(69)^. The circulating concentrations of PUFA fluctuate throughout gestation with the placenta controlling the uptake and transfer of maternal PUFA to the fetus^(70)^. Previous evidence has suggested that placental uptake and transfer differ, with the transfer favouring DHA and AA, particularly during the last trimester of pregnancy. The differing associations apparent within the current study may be owing to differences in genetics influencing PUFA metabolism, in addition to the altered fatty acid metabolism throughout pregnancy; the maternal PUFA status reflects exposure throughout the entire pregnancy, whilst the cord PUFA status may more accurately depict exposure throughout the last trimester. The placenta has been shown to play an active role in fetal programming^(72)^, and given that PUFA transfer across the placenta differs throughout gestation, it may point towards specific periods during pregnancy where the developing fetus may be particularly sensitive to the effects of the *n*-3 and *n*-6 PUFA. In future, a focused examination using repeat measurements of PUFA status throughout pregnancy may help to elucidate the differing associations between the maternal and cord PUFA and child adiposity.

The current study has several strengths. First, the use of robust biological measures of both cord and maternal PUFA concentrations and the assessment of dietary intakes of fish provide a robust depiction of exposures throughout pregnancy. The use of numerous time points also helped to determine if associations persisted throughout childhood. The large cohort size and the high participant retainment throughout the follow-up appointments are also particular strengths of this research. Finally, this cohort was well characterised at numerous time points, allowing the comprehensive adjustment of confounders, including fish intakes throughout childhood. There are, however, several limitations. Like all observational epidemiological studies, no cause-and-effect relationships can be established and can only identify associations, which might be non-causal. Although models were adjusted for a range of key maternal and child characteristics, we cannot exclude the possibility of residual confounding from unmeasured factors. Variables such as gestational weight gain, paternal BMI, maternal physical activity, broader dietary patterns or other environmental exposures may have influenced child body weight and should be considered when interpreting these findings. These variables were not available as the present study was a secondary analysis of an existing cohort that was not originally designed to address these specific outcomes. The practical challenges of conducting data collection in a developing country also limited the feasibility of capturing all potential confounders. Maternal fish intakes were assessed using an FUQ administered at 28 weeks’ gestation which may not accurately capture fish consumption across the entire pregnancy. Moreover, the FUQ cannot capture other dietary factors which may have influenced results. Additionally, maternal PUFA status was measured at one time point only, and as mentioned, future research may wish to use repeated measures to determine any sensitive periods as to when the developing fetus may be differentially susceptible to the effects of the *n*-3 and *n*-6 PUFA. We were also unable to assess PUFA status throughout childhood. It is worth noting that in the present study, fatty acids were reported as absolute concentrations rather than as percentages of total fatty acids which may limit comparability to other studies. During pregnancy, however, absolute concentrations may more closely reflect maternal–fetal exposure, as placental transfer depends on circulating levels. In contrast, percentage values can be affected by changes in other lipid fractions, which fluctuate substantially in pregnancy^(73)^. Finally, the methods used were to enable the detection of subtle associations between exposures and measures of child weight, and thus, the clinical significance of these findings is unclear.

### Conclusions

In summary, in this high fish-eating cohort, maternal total fish consumption throughout pregnancy was not associated with child weight at birth or throughout childhood. This study, however, suggests that lower cord DHA, an *n*-3 PUFA, may be associated with higher risk of LGA at birth, whilst higher *n*-6 PUFA during pregnancy may be associated with adiposity development throughout childhood to early adolescence. This study has global relevance given the transition towards westernized-style diets, typically higher in *n*-6 PUFA and lower in *n*-3 PUFA, in many countries. Future research is needed to explore the potential long-term metabolic consequences of these associations.

## Supplementary Material

1

**Supplementary material.** To view supplementary material for this article, please visit https://doi.org/10.1017/S0007114526106369

## Figures and Tables

**Table 1.** Maternal and child characteristics of study population (n = 1185)

	*n*	Mean	SD	Median	IQR	Minimum	Maximum
Maternal age (years)	1185	27·1	6·3	26·1	22·1, 31·4	16·3	46·8
Maternal BMI (kg/m^2^)	1185	26·9	6·6	26·1	21·8, 31·0	14·7	49·6
Total fish consumption (g/d)	1124	118·0	78·8	100·0	71·4, 145·7	0·0	584·7
Hollingshead socio-economic status	1185	32·2	10·3	31·5	24·0, 39·5	14·0	63·0
Maternal total *n*-3 PUFA (mg/ml)	1185	0·27	0·09	0·26	0·20, 0·33	0·12	0·64
Maternal total *n*-6 PUFA (mg/ml)	1185	1·10	0·29	1·08	0·89, 1·30	0·43	2·71
Gestational age (weeks)	1185	39·1	1·4	39·0	38·0, 40·0	34·0	41·0
Birth weight (kg)	1185	3·19	0·46	3·20	2·90, 3·49	1·62	5·20
Child weight at 20 months (kg)	1182	11·3	1·6	11·1	10·1, 12·1	7·1	20·3
Child weight at 7 years (kg)	1167	26·3	6·7	24·5	21·9, 28·9	13·0	64·6
Child weight at 13 years (kg)	877	54·4	16·3	50·5	42·6, 63·0	24·5	134·5

.IQR, interquartile range..

**Table 2.** Associations between maternal fish intakes and birth weight

		SGA			LGA		
		OR	95 % CI	*P*	OR	(95 % CI)	*P*
Total fish	>145·71	Ref	–	–	Ref	–	–
	100·00–145·70	1·07	0·70–1·64	0·753	0·79	0·34–1·78	0·563
	71·42–99·99	1·42	0·90–2·25	0·130	0·43	0·13–1·37	0·152
	< 71·41	1·01	0·64–1·60	0·958	0·58	0·22–1·51	0·265
Lean fish	>57·14	Ref	–	–	Ref	–	–
	28·57–57·13	0·83	0·54–1·29	0·409	0·47	0·18–1·22	0·167
	0·01–28·56	1·43	0·85–2·39	0·176	0·41	0·90–1·82	0·238
	0·00	0·83	0·56–1·23	0·357	0·57	0·26–1·26	0·167
Oily fish	>100·00	Ref	–	–	Ref	–	–
	71·42–99·99	0·96	0·61–1·51	0·853	0·30	0·09–1·07	0·064
	34·28–71·41	0·99	0·64–1·51	0·948	0·70	0·29–1·64	0·407
	< 34·27	1·22	0·80–1·85	0·351	0·82	0·35–1·94	0·653

SGA, small for gestational age; LGA, large for gestational age.

Adjusted for maternal age, maternal BMI, Hollingshead socio-economic status, gestational age, child sex, parity and alcohol use.

**Table 3.** Associations between maternal fish intakes (g/d) and risk of overweight/obesity throughout childhood

		20 months Overweight/obesity			7 years Overweight/obesity			13 years Overweight/obesity		
		OR	95 % CI	*P*	OR	95 % CI	*P*	OR	95 % CI	*P*
Total fish	< 71·41	Ref	–	–	Ref	–	–	Ref	–	–
	71·42–99·99	1·38	0·96–2·00	0·082	0·91	0·55–1·49	0·620	1·11	0·64–1·94	0·711
	100·00–145·70	1·28	0·92–1·79	0·141	1·03	0·70–1·72	0·894	1·36	0·83–2·11	0·218
	>145·71	1·13	0·80–1·61	0·490	1·10	0·70–1·73	0·952	1·22	0·73–2·04	0·456
Lean fish	0·00	Ref	–	–	Ref	–	–	Ref	–	–
	0·01–28·56	1·17	0·77–1·79	0·457	1·02	0·56–1·80	0·951	1·66	0·87–3·17	0·126
	28·57–57·13	0·87	0·63–1·22	0·425	0·83	0·54–1·27	0·394	1·54	0·96–2·47	0·074
	>57·14	1·32	0·63–1·78	0·070	1·09	0·74–1·60	0·677	**1·69**	**1·09–2·62**	**0·020**
Oily fish	< 34·27	Ref	–	–	Ref	–	–	Ref	–	–
	34·28–71·41	0·90	0·64–1·28	0·568	1·17	0·74–1·84	0·512	0·92	0·55–1·53	0·747
	71·42–99·99	1·17	0·81–1·69	0·394	1·19	0·73–1·95	0·493	1·17	0·67–2·01	0·579
	>100·00	1·02	0·74–1·42	0·893	1·12	0·72–1·76	0·610	0·73	0·44–1·21	0·227

Adjusted for maternal age, maternal BMI, Hollingshead socio-economic status, gestational age, child sex, parity and alcohol. Models at 7 and 13 years additionally adjusted for child fish intakes.

**Table 4.** Associations between maternal PUFA (mg/ml) and risk of overweight/obesity throughout childhood

		20 months Overweight/obesity			7 years Overweight/obesity			13 years Overweight/obesity		
		OR	95 % CI	*P*	OR	95 % CI	*P*	OR	95 % CI	*P*
LA	<0·723	Ref	–	–	Ref	–	–	Ref	–	–
	0·723–0·884	1·35	0·97–1·90	0·079	1·17	0·74–1·84	0·511	1·26	0·76–2·07	0·367
	0·885–1·049	**1·51**	**1·08–2·12**	**0·017**	1·54	0·98–2·42	0·061	**1·83**	**1·11–3·02**	**0·018**
	>1·050	**1·45**	**1·03–2·03**	**0·032**	**1·61**	**1·03–2·53**	**0·037**	**2·13**	**1·29–3·52**	**0·003**
AA	<0·147	Ref	–	–	Ref	–	–	Ref	–	–
	0·148–0·204	0·84	0·60–1·17	0·291	1·33	0·81–2·17	0·255	0·90	0·53–1·53	0·693
	0·205–0·260	**0·68**	**0·48–0·95**	**0·023**	1·13	0·69–1·85	0·634	1·29	0·76–2·20	0·351
	>0·261	0·84	0·60–1·17	0·293	1·30	0·80–2·12	0·297	1·55	0·91–2·65	0·105
Total *n*-6	<0·895	Ref	–	–	Ref	–	–	Ref	–	–
	0·896–1·090	1·29	0·92–1·82	0·138	1·14	0·71–1·82	0·597	0·90	0·54–1·49	0·671
	1·091–1·296	**1·63**	**1·16–2·28**	**0·005**	1·43	0·91–2·24	0·125	1·15	0·70–1·91	0·576
	>1·297	1·26	0·90–1·77	0·183	**1·61**	**1·03–2·52**	**0·039**	**2·10**	**1·29–3·42**	**0·003**
ALA	<0·0348	Ref	–	–	Ref	–	–	Ref	–	–
	0·0349–0·0350	1·11	0·80–1·55	0·532	1·15	0·74–1·79	0·535	1·26	0·76–2·08	0·367
	0·0351–0·0379	1·13	0·79–1·62	0·513	1·08	0·68–1·74	0·738	1·18	0·69–2·02	0·557
	>0·0380	0·87	0·61–1·25	0·454	1·17	0·74–1·85	0·490	1·20	0·73–1·98	0·469
EPA	<0·0488	Ref	–	–	Ref	–	–	Ref	–	–
	0·0488–0·0489	1·20	0·80–1·81	0·382	0·75	0·45–1·24	0·261	0·96	0·55–1·68	0·882
	0·0490–0·0513	1·31	0·87–1·96	0·198	0·96	0·59–1·56	0·857	1·41	0·82–2·44	0·220
	>0·0514	1·06	0·69–1·62	0·792	0·74	0·44–1·24	0·257	1·06	0·60–1·87	0·837
DHA	<0·112	Ref	–	–	Ref	–	–	Ref	–	–
	0·113–0·178	1·20	0·87–1·67	0·269	1·07	0·66–1·74	0·773	0·72	0·41–1·27	0·262
	0·179–0·243	0·76	0·54–1·07	0·112	0·93	0·58–1·50	0·774	1·05	0·61–1·80	0·869
	>0·244	0·74	0·52–1·04	0·081	0·95	0·59–1·55	0·850	1·47	0·85–2·54	0·168
Total n-3	<0·198	Ref	–	–	Ref	–	–	Ref	–	–
	0·199–0·264	1·28	0·92–1·78	0·142	1·22	0·75–1·97	0·442	0·81	0·46–1·43	0·467
	0·265–0·336	0·80	0·87–1·12	0·195	0·87	0·54–1·41	0·580	1·09	0·64–1·87	0·747
	>0·337	0·73	0·52–1·03	0·075	0·92	0·56–1·50	0·736	1·47	0·85–2·55	0·172
*n*6:*n*3	<3·272	Ref	–	–	Ref	–	–	Ref	–	–
	3·273–3·943	1·34	0·95–1·89	0·091	1·35	0·86–2·11	0·188	1·28	0·79–2·05	0·314
	3·944–5·010	**1·42**	**1·01–1·99**	**0·045**	1·43	0·92–2·23	0·109	1·27	0·79–2·02	0·325
	>5·011	**1·67**	**1·19–2·35**	**0·003**	**1·75**	**1·07–2·85**	**0·025**	1·23	0·70–2·16	0·469

LA, linoleic acid; AA, arachidonic acid; ALA, alpha-linolenic acid.

Adjusted for maternal age, maternal BMI, Hollingshead socio-economic status, gestational age, child sex, parity, alcohol. Models at 7 and 13 years additionally adjusted for child fish intakes....

**Table 5.** Associations between cord PUFA (mg/ml) and birth weight

PUFA (mg/ml)		SGA		*P*	LGA		*P*
		OR	95 % CI		OR	95 % CI	
**Cord LA**	>0·189	Ref	–	–	Ref	–	–
	0·158–0·188	0·86	0·53–1·40	0·539	0·30	0·06–1·49	0·141
	0·115–0·147	0·85	0·52–1·40	0·529	0·93	0·31–2·76	0·894
	<0·114	0·71	0·42–1·19	0·192	1·99	0·77–5·17	0·158
**Cord AA**	>0·266	Ref	–	–	Ref	–	–
	0·190–0·265	0·97	0·59–1·61	0·911	0·91	0·30–2·79	0·867
	0·145–0·189	0·96	0·57–1·56	0·864	0·91	0·31–2·69	0·865
	<0·144	1·02	0·62–1·70	0·932	1·26	0·45–3·51	0·662
**Cord total *n*-6**	>0·514	Ref	–	–	Ref	–	–
	0·365–0·513	0·80	0·48–1·34	0·391	0·69	0·21–2·23	0·551
	0·277–0·364	1·10	0·67–1·80	0·708	0·85	0·28–2·62	0·781
	<0·276	0·94	0·56–1·56	0·807	1·67	0·63–4·41	0·299
**Cord DHA**	>0·128	Ref	–	–	Ref	–	–
	0·097–0·127	0·83	0·50–1·39	0·484	1·93	0·54–6·83	0·309
	0·072–0·096	0·82	0·49–1·37	0·445	1·61	0·44–5·92	0·476
	<0·071	1·03	0·63–1·70	0·906	**4·17**	**1·30–13·44**	**0·017**
**Cord *n*6: *n*3**	>5·587	Ref	–	–	Ref	–	–
	3·349–5·586	1·00	0·62–1·62	0·993	1·09	0·39–3·01	0·875
	2·590–3·348	**0·54**	**0·31–0·93**	**0·027**	1·19	0·46–3·06	0·722
	<2·589	0·99	0·60–1·61	0·955	0·33	0·08–1·27	0·105

SGA, small for gestational age; LGA, large for gestational age; LA, linoleic acid; AA, arachidonic acid.

Adjusted for maternal age, maternal BMI, Hollingshead socio-economic status, gestational age, child sex, parity and alcohol use.

## Abbreviations:

AA: arachidonic acid
FUQ: Fish Use Questionnaire
LA: linoleic acid
LGA: large for gestational age
SGA: small for gestational age

## References

[1] Stephenson J, Heslehurst N, Hall J, (2018) Before the beginning: nutrition and lifestyle in the preconception period and its importance for future health. Lancet 391(10132), 1830–1841. 29673873 10.1016/S0140-6736(18)30311-8 PMC6075697

[2] Barker DJ (1995) Fetal origins of coronary heart disease. Br Med J 311(6998), 171–174. 7613432 10.1136/bmj.311.6998.171 PMC2550226

[3] Almond D & Currie J (2011) Killing me softly: the fetal origins hypothesis. J Econ Perspec 25(3), 153–172. 10.1257/jep.25.3.153 PMC4140221 25152565

[4] Min M, Wang S-F, Sheng J, (2012) Birth weight and subsequent blood pressure: a meta-analysis. Arch Cardiovasc Dis 105(2), 99–113. 22424328 10.1016/j.acvd.2011.10.006

[5] Knop MR, Geng T-T, Gorny AW, (2018) Birth weight and risk of type 2 diabetes mellitus, cardiovascular disease, and hypertension in adults: a meta-analysis of 7,646,267 participants from 135 studies. J Am Heart Assoc 7(23), e008870. 30486715 10.1161/JAHA.118.008870 PMC6405546

[6] Raisi-Estabragh Z, Cooper J, Bethell MS, (2023) Lower birth weight is linked to poorer cardiovascular health in middle-aged population-based adults. Heart 109, 535–541. 36384749 10.1136/heartjnl-2022-321733 PMC10086465

[7] Orsso C, Colin-Ramirez E, Field C, Madsen K, (2020) Adipose tissue development and expansion from the womb to adolescence: an overview. Nutrients 12(9), 1–21. 10.3390/nu12092735 PMC7551046 32911676

[8] Palacios-Marin I, Serra D, Jimenez-Chillarion J, Herrero L, (2023) Childhood obesity: implications on adipose tissue dynamics and metabolic health. Obes Rev 24(12), e13627. 37608466 10.1111/obr.13627

[9] Meher A, Randhir K, Mehendale S, Wagh G, (2016) Maternal fatty acids and their association with birth outcome: a prospective study. PLoS One 11(1), e0147359. 26815428 10.1371/journal.pone.0147359 PMC4729437

[10] Grootendorst-van M, Tiemeier H, Steenweg-de Graaff J, (2018) Maternal plasma n-3 and n-6 polyunsaturated fatty acids during pregnancy and features of fetal health: fetal growth velocity, birth weight and duration of pregnancy. Clin Nutr 37, 1367–74. 28651830 10.1016/j.clnu.2017.06.010

[11] Abdelraham MA, Osama H, Saeed H, (2023) Impact of n-3 polyunsaturated fatty acid intake in pregnancy on maternal health and birth outcomes: systematic review and meta-analysis from randomized controlled trials. Arch Gynecol Obstet 307(1), 249–262. 35348829 10.1007/s00404-022-06533-0

[12] Bergmann RL, Bergmann KE, Haschke-Becher E, (2007) Does maternal docosahexaenoic acid supplementation during pregnancy and lactation lower BMI in late infancy. J Perinat Med 35(4), 295–300. 17547539 10.1515/JPM.2007.085

[13] Firouzabadi FD, Shab-Bidar S & Jayedi A (2022) The effects of omega-3 polyunsaturated fatty acids supplementation in pregnancy, lactation, and infancy: an umbrella review of meta-analyses of randomized trials. Pharmacol Res 177, 106100. 35104631 10.1016/j.phrs.2022.106100

[14] Fleckenstein-Elsern M, Dinnies D, Jelenik T, (2016) Eicosapentaenoic acid and arachidonic acid differentially regulate adipogenesis, acquisition of a brite phenotype and mitochondrial function in primary human adipocytes. Mol Nutr Food Res 60(9), 2065–2075. 27159788 10.1002/mnfr.201500892

[15] Hao L, Chen C, Nie Y, (2023) Differential interventional effects of omega-6 and omega-3 polyunsaturated fatty acids on high fat diet-induced obesity and hepatic pathology. Int J Mol Sci 24(24), 17261. 38139090 10.3390/ijms242417261 PMC10743920

[16] Bernard J, Tint M, Aris I, (2017) Maternal plasma phosphatidylcholine polyunsaturated fatty acids during pregnancy and offspring growth and adiposity. Prostaglandins, Leukotrienes, Essent Fatty Acids 121, 21–29. 10.1016/j.plefa.2017.05.006 PMC5501311 28651694

[17] Wu S, Zhao F, He Y, (2022) Association between maternal erythrocyte PUFA levels during pregnancy and offspring weight status: a birth cohort study. Front Nutr 9, 978679. 36245520 10.3389/fnut.2022.978679 PMC9557224

[18] Al-Hinai M, Baylin A, Tellez-Rojo M, (2018) Maternal intake of omega-3 and omega-6 polyunsaturated fatty acid during mid-pregnancy is inversely associated with linear growth. J Dev Orig Health Dis 9(4), 432–441. 29665872 10.1017/S2040174418000193 PMC6716373

[19] Donahue S, Rifas-Shiman S, Gold D, (2011) Prenatal fatty acid status and child adiposity at age 3 y: results from a US pregnancy cohort. Am J Clin Nutr 92(4), 780–788. 10.3945/ajcn.110.005801 PMC3057547 21310834

[20] Pereira-da-Silva L, Cabo C, Moreira A, (2015) The effect of long chain polyunsaturated fatty acids intakes during pregnancy on adiposity of healthy full-term offspring at birth. J Perinatol 35(3), 177–189. 25321648 10.1038/jp.2014.188

[21] Larque E, Pagan A, Prieto MT, (2014) Placental fatty acid transfer: a key factor in fetal growth. Ann Nutr Metab 64(3–4), 247–253. 25300267 10.1159/000365028

[22] Mendivil CO (2021) Fish consumption: a review of its effects on metabolic and hormonal health. Nutr Metab Insights 14, 1. 10.1177/11786388211022378 PMC8182174 34158802

[23] Scientific Advisory Committee on Nutrition (2004) Advice on fish consumption: benefits and risks 2004. TSO, United Kingdom.

[24] USFDA (2021). Advice about eating fish. Available from https://www.fda.gov/media/102331/download.

[25] Leventakou V, Roumeliotaki T, Martinez D, (2014) Fish intake during pregnancy, fetal growth, and gestational length in 19 European birth cohort studies. Am J Clin Nutr 99(3), 506–516. 24335057 10.3945/ajcn.113.067421

[26] Wei Z, Li W, Lei C, (2023) Maternal seafood consumption and fetal growth: a birth cohort study in urban China. BMC Pregnancy Childbirth 23, 253. 37055723 10.1186/s12884-023-05431-w PMC10099888

[27] Van den Berg S, Wijga A, van Rossem L, (2016) Maternal fish consumption during pregnancy and BMI in children from birth up to age 14 years: the PIAMA cohort study. European J Nutr 55(2), 799–808. 25893718 10.1007/s00394-015-0901-6

[28] Strain JJ, Yeates AJ, van Wijngaarden E, (2015) Prenatal exposure to methyl mercury from fish consumption and polyunsaturated fatty acids: associations with child development at 20 months of age in an observational study in the Republic of Seychelles. Am J Clin Nutr 101(3), 530–537. 25733638 10.3945/ajcn.114.100503 PMC4340059

[29] Wesolowska M, Yeates AJ, McSorley EM, (2024) Dietary selenium and mercury intakes from fish consumption during pregnancy: Seychelles child development study nutrition cohort 2. Neurotoxicology 101, 1–5. 38135192 10.1016/j.neuro.2023.12.012

[30] Conway M, Mulhern M, McSorley E, (2018) Dietary determinants of polyunsaturated fatty acid (PUFA) status in a high fish-eating cohort during pregnancy. Nutrients 10, 927. 30036954 10.3390/nu10070927 PMC6073891

[31] Folch J, Lees M & Sloane Stanley GH (1957) A simple method for the isolation and purification of total lipides from animal tissue. J Biol Chem 226(1), 497–509. 13428781

[32] World Health Organisation (WHO) Child Growth Standards (2006) Available at: https://www.who.int/tools/child-growth-standards.

[33] De Onis M & Lobstein T (2010) Defining obesity risk status in the general childhood population: which cut-offs should we use? Int J Paediatric Obes 5, 458–460. 10.3109/17477161003615583 20233144

[34] Van Wijngaarden E, Harrington D, Kobrosly R, (2014) Prenatal exposure to methylmercury and LCPUFA in relation to birth weight. Ann Epidemiol 24(4), 273–8. 24525104 10.1016/j.annepidem.2014.01.002 PMC3951518

[35] Yeates AJ, Zavez A, Thurston SW, (2020) Maternal long-chain polyunsaturated fatty acid status, methylmercury exposure, and birth outcomes in a high-fish-eating mother-child cohort. J Nutr 150(7), 1749–1756. 32433731 10.1093/jn/nxaa131 PMC7330473

[36] Stratakis N, Roumeliotaki T, Oken O, (2016) Fish intake in pregnancy and child growth: a pooled analysis of 15 European and US Birth cohorts. JAMA Pediatr 170(4), 381–390. 26882542 10.1001/jamapediatrics.2015.4430 PMC5103635

[37] Davidson PW, Myers GJ, Cox C, (1998) Effects of prenatal and postnatal methylmercury exposure from fish consumption on neurodevelopment. JAMA 280, 701–7. 9728641 10.1001/jama.280.8.701

[38] Davidson PW, Strain JJ, Myers GJ, (2008) Neurodevelopmental effects of maternal nutritional status and exposure to methylmercury from eating fish during pregnancy. Neurotoxicol 29(5), 767–775. 10.1016/j.neuro.2008.06.001 PMC2580738 18590763

[39] Guldner L, Monfort C, Rouget F, (2007) Maternal fish and shellfish intake and pregnancy outcomes: a prospective cohort study in Brittany, France. Environ Health 6(1), 33. 17958907 10.1186/1476-069X-6-33 PMC2211746

[40] Mohanty AF, Thompson M, Burbacher TM, (2015) Periconceptional seafood intake and fetal growth. Paediatric Perinat Epidemiol 25(5), 376–387. 10.1111/ppe.12205 PMC4536156 26147526

[41] Brantsaeter AL, Birgisdottir BE, Meltzer HM, (2012) Maternal seafood consumption and infant birth weight, length, and head circumference in the Norwegian mother and child cohort study. Br J Nutr 107(3), 436–444. 21767447 10.1017/S0007114511003047

[42] Willer DF, Newton R, Malcorps W, (2024) Wild fish consumption can balance nutrient retention in farmed fish. Nat Food 5, 221–229. 38509235 10.1038/s43016-024-00932-z PMC10963266

[43] Clemente-Suarewz VJ, Beltran-Velasco AI, Redondo-Florez L, (2023) Global impacts of western diet and its effects of metabolism and health: a narrative review. Nutrients 15(12), 2749. 37375654 10.3390/nu15122749 PMC10302286

[44] Cardoso I, Bovet P, Viswanathan B, Luke A, (2013) Nutrition transition in a middle-income country: 22-year trends in the Seychelles. Eur J Clin Nutr 67(2), 135–140. 23249880 10.1038/ejcn.2012.199 PMC3786399

[45] Makrides M (2011) The role of n-3 LCUFA in pregnancy. Lipids Brain 18(5), 255–258.

[46] Olsen SF, Halldorsson TI, Thorne-Lyman AI, (2018) Plasma concentrations of long chain n-3 fatty acids in early and mid-pregnancy and risk of early preterm birth. EBioMedicine 25, 325–333. 10.1016/j.ebiom.2018.07.009 PMC6156714 30082226

[47] Vidakovic A, Gishti O, Voortman T, (2016) Maternal plasma PUFA concentrations during pregnancy and childhood adiposity: the generation R study. Am J Clin Nutr 103(4), 1017–1025. 26912493 10.3945/ajcn.115.112847 PMC5426536

[48] Moon R, Harvey N, Robinson S, (2013) Maternal plasma polyunsaturated fatty acid in late pregnancy is associated with offspring body composition in childhood. J Clin Endocrin Metab 98(1), 299–307. 10.1210/jc.2012-2482 PMC3604685 23162098

[49] Solorzano CMB & McCartney CR (2010) Obesity and the pubertal transition in girls and boys. Reprod 140(3), 399–410. 10.1530/REP-10-0119 PMC2931339 20802107

[50] Yeates AJ, Love TM, Engström K, (2015) Genetic variation in FADS genes is associated with maternal long-chain PUFA status but not with cognitive development of infants in a high fish-eating observational study. Prostaglandins Leukot Essent Fatty Acids 102–103, 13–20. 10.1016/j.plefa.2015.08.004 PMC4746501 26474818

[51] De Vries PS, Gielen M, Rizopoulos D, (2014) Association between polyunsaturated fatty acid concentrations in maternal plasma phospholipids during pregnancy and offspring adiposity at age 7: The MEFAB cohort. Prostaglandins, Leukotrienes Essent Fatty Acids 91(3), 81–85. 10.1016/j.plefa.2014.04.002 24813643

[52] Calder PC (2020) n-3 PUFA and inflammation: from membrane to nucleus and from bench to bedside. Proc Nutr Soc 79, 404–416. 10.1017/S0029665120007077 32624016

[53] Tang X, Hou Y, Schwartz TW & Haeggstrom JZ (2022) Metabolic G-protein coupled receptor signalling: potential regulation of eicosanoids. Biochem Pharmacol 204, 115208. 35963340 10.1016/j.bcp.2022.115208

[54] Majed BH & Khalil RA (2012) Molecular mechanisms regulating the vascular prostacyclin pathways and their adaptation during pregnancy and in the newborn. Pharmacol Rev 63(3), 540–582. 10.1124/pr.111.004770 PMC3400831 22679221

[55] Rahaman MS (2018) Prostacyclin: a major prostaglandin in the regulation of adipose tissue development. J Cell Physiol 234(4), 3254–3262. 30431153 10.1002/jcp.26932

[56] Kabaran S & Besler HT (2015) Do fatty acids affect fetal programming?. J Health Popul Nutr 33, 14. 26825664 10.1186/s41043-015-0018-9 PMC5025983

[57] Orsavova J, Misurcova L, Ambrozova JV, (2015) Fatty acids composition of vegetable oils and its contribution to dietary energy intake and dependence of cardiovascular mortality on dietary intake of fatty acids. Int J Mol Sci 16(6), 12871–90. 26057750 10.3390/ijms160612871 PMC4490476

[58] Mariamenatu AH & Abdu EM (2021) Overconsumption of omega-6 polyunsaturated fatty acids (PUFAs) versus deficiency of omega-3 PUFAs in modern-day diets: the disturbing factor for their “balanced antagonistic metabolic functions” in the human body. J Lipids 2021, 8848161. 33815845 10.1155/2021/8848161 PMC7990530

[59] Liput KP, Lepczyński A, Ogłuszka M, (2021) Effects of dietary n-3 and n-6 polyunsaturated fatty acids in inflammation and cancerogenesis. Int J Mol Sci 22(13), 6965. 34203461 10.3390/ijms22136965 PMC8268933

[60] Monaco-Brown M & Lawrence DA (2022) Obesity and maternal-placental-fetal immunology and health. Front Pediatr 10, 859885. 35573953 10.3389/fped.2022.859885 PMC9100592

[61] Baker EJ, Calder PC, Kermack AJ, (2024) Omega-3 LC-PUFA consumption is now recommended for women of childbearing age and during pregnancy to protect against preterm and early preterm birth: implementing this recommendation in a sustainable manner. Front Nutr 11, 1502866. 39677502 10.3389/fnut.2024.1502866 PMC11639083

[62] Cinelli G, Fabrizi M, Ravà L, (2018) Association between maternal and foetal erythrocyte fatty acid profiles and birth weight. Nutrients 10, 402. 29570689 10.3390/nu10040402 PMC5946187

[63] Kilari A, Mehendale S, Dangat K, (2010) Associations of long-chain polyunsaturated fatty acid concentrations with birth outcomes in term Indian mothers and their neonates. Am J Hum Biol 23(3), 319–324. 21484911 10.1002/ajhb.21129

[64] Calder PC (2012) Mechanisms of action of (n-3) fatty acids. J Nutr 142(3), 592S–599S. 22279140 10.3945/jn.111.155259

[65] Albracht-Schlulte K, Kalupahana NS, Ramalingam L, (2018) Omega-3 fatty acids in obesity and metabolic syndrome: a mechanistic update. Biochemistry 58, 1–16. 10.1016/j.jnutbio.2018.02.012 PMC7561009 29621669

[66] Sridhar SB, Ferrara A, Ehrlich SF, (2013) Risk of large-for-gestational-age newborns in women with gestational diabetes by race and ethnicity and body mass index categories. Obstet Gynecol 121(6), 1255–1262. 23812460 10.1097/AOG.0b013e318291b15c PMC5079180

[67] Younes S, Samara M, Salama N, (2021) Incidence, risk factors, and feto-maternal outcomes of inappropriate birth weight for gestational age among singleton live births in Qatar: a population-based study. PLoS One 16(10), e025896. 10.1371/journal.pone.0258967 PMC8553085 34710154

[68] Hong YH & Lee JE (2021) Large for gestational age and obesity-related comorbidities. J Obes Metab Syndr 30(2), 124–131. 34053939 10.7570/jomes20130 PMC8277589

[69] Hanebutt FL, Demmelmair H, Schiessl B, (2008) Long-chain polyunsaturated fatty acid (LC-PUFA) transfer across the placenta. Clin Nutr 27(5), 685–693. 18639956 10.1016/j.clnu.2008.05.010

[70] Duttaroy AK (2009) Transport of fatty acids across the human placenta: a review. Prog Lipid Res 48(1), 52–61. 19041341 10.1016/j.plipres.2008.11.001

[71] Haggarty P (2010) Fatty acid supply to the human fetus. Annu Rev Nutr 30, 237–255. 20438366 10.1146/annurev.nutr.012809.104742

[72] Kwon EJ & Kim YJ (2017) What is fetal programming? A lifetime health is under the control of in utero health. Obster Gynecol Sc0 60(6), 506–519. 10.5468/ogs.2017.60.6.506 PMC5694724 29184858

[73] Brenna JT, Plourde M, Stark KD, (2018) Best practices for the design, laboratory analysis, and reporting of trials involving fatty acids. Am J Clin Nutr 108(2), 211–227. 29931035 10.1093/ajcn/nqy089 PMC6084616

