# Supplementary material for: Associations between maternal fish intakes, maternal and cord PUFA and longitudinal measures of child weight at birth, 20 months and at 7 and 13 years of age
Source: Br J Nutr. 2026 Feb 5;135(10):1048–58. doi: 10.1017/S0007114526106369 (PMC13058842; doi:10.1017/S0007114526106369)
Supplement: Mcmullan et al. supplementary material [file S0007114526106369sup001.docx]

| **Supplementary Table 1: Maternal (28 weeks gestation) and cord PUFA concentrations (mg/ml)** | | | | | | |
| --- | --- | --- | --- | --- | --- | --- |
|  | **Mean** | **SD** | **Median** | **IQR** | **Minimum** | **Maximum** |
| *Maternal (28 weeks gestation) (n = 1185)* |  |  |  |  |  |  |
| LA | 0.90 | 0.25 | 0.88 | 0.72, 1.05 | 0.38 | 2.33 |
| AA | 0.20 | 0.08 | 0.20 | 0.15, 0.26 | 0.04 | 0.38 |
| Total n-6 | 1.10 | 0.08 | 1.08 | 0.89, 1.30 | 0.43 | 2.70 |
| ALA | 0.037 | 0.006 | 0.035 | 0.035, 0.038 | 0.000 | 0.114 |
| EPA | 0.052 | 0.008 | 0.049 | 0.049, 0.050 | 0.049 | 0.116 |
| DHA | 0.18 | 0.08 | 0.18 | 0.12, 0.24 | 0.04 | 0.52 |
| Total n-3 | 0.27 | 0.09 | 0.26 | 0.20, 0.33 | 0.12 | 0.64 |
| n6:n3 | 4.36 | 1.60 | 3.97 | 3.29, 5.00 | 1.56 | 15.82 |
| *Cord blood* (n = 932)* |  |  |  |  |  |  |
| LA | 0.16 | 0.11 | 0.15 | 0.12, 0.19 | 0.01 | 1.73 |
| AA | 0.28 | 0.23 | 0.19 | 0.15, 0.28 | 0.03 | 1.18 |
| Total n-6 | 0.45 | 0.26 | 0.37 | 0.28, 0.52 | 0.10 | 2.14 |
| DHA | 1.02 | 0.05 | 0.10 | 0.07, 0.13 | 0.01 | 0.55 |
| n6:n3 | 5.48 | 5.70 | 3.38 | 2.62, 5.90 | 1.02 | 47.71 |
| **Cord ALA and EPA below the lower limit of detection*  *SD; Standard deviation, IQR; Interquartile range, PUFA; Polyunsaturated fatty acids, ALA; alpha-linolenic acid, DHA; docosahexaenoic acid, EPA; eicosapentaenoic acid, LA; linoleic acid*  *Total n-3 PUFA is defined as the sum of ALA + EPA + DHA; Total n-6 PUFA is defined as the sum of LA + AA.* | | | | | | |

| **Supplementary Table 2: Associations between maternal PUFA (mg/ml) and birth weight** | | | | | | | | |
| --- | --- | --- | --- | --- | --- | --- | --- | --- |
|  | | **SGA** | | |  | **LGA** | | |
| **PUFA (mg/ml)** | | **OR** | **95% CI** | **P-value** |  | **OR** | **95% CI** | **P-value** |
| **LA** | > 1.050  0.885-1.049  0.723-0.884  < 0.723 | Ref  1.28  1.20  1.31 | -  0.82-1.98  0.77-1.87  0.85-2.03 | -  0.280  0.413  0.280 |  | Ref  1.45  0.93  0.62 | -  0.62-3.41  0.37-2.36  0.22-1.76 | -  0.389  0.882  0.373 |
| **AA** | >0.261  0.205-0.260  0.149-0.204  < 0.148 | Ref  1.08  1.17  1.18 | -  0.69-1.68  0.76-1.81  0.76-1.82 | -  0.736  0.475  0.455 |  | Ref  1.01  0.93  **0.26** | -  0.45-2.29  0.39-2.20  **0.07-0.94** | -  0.977  0.870  **0.041** |
| **Total n-6** | >1.297  1.091-1.296  0.896-1.090  < 0.895 | Ref  1.05  1.03  1.26 | -  0.67-1.63  0.66-1.59  0.82-1.94 | -  0.840  0.901  0.284 |  | Ref  1.18  0.70  0.44 | -  0.52-2.66  0.28-1.76  0.15-1.28 | -  0.692  0.452  0.133 |
| **ALA** | >0.0380  0.0351-0.0379  0.0349-0.0350  < 0.0348 | Ref  0.78  0.90  1.22 | -  0.49-1.24  0.60-1.35  0.79-1.89 | -  0.296  0.607  0.369 |  | Ref  0.63  0.63  0.88 | -  0.35-2.20  0.27-1.45  0.35-2.21 | -  0.339  0.274  0.789 |
| **EPA** | >0.0514  0.0491-0.0513  0.0489-0.0490  < 0.0488 | Ref  0.97  1.21  0.81 | -  0.65-1.47  0.81-1.82  0.46-1.43 | -  0.896  0.351  0.474 |  | Ref  0.78  0.48  0.32 | -  0.36-1.68  0.20-1.17  0.07-1.45 | -  0.516  0.107  0.135 |
| **DHA** | >0.244  0.179-0.243  0.114-0.178  < 0.113 | Ref  1.05  1.34  1.24 | -  0.67-1.64  0.87-2.06  0.80-1.93 | -  0.336  0.192  0.336 |  | Ref  1.06  0.78  0.91 | -  0.44-2.52  0.29-2.07  0.36-2.29 | -  0.904  0.614  0.845 |
| **Total n-3** | > 0.337  0.265-0.336  0.199-0.264  < 0.198 | Ref  1.11  1.28  1.24 | -  0.71-1.73  0.83-1.98  0.80-1.93 | -  0.660  0.267  0.337 |  | Ref  1.07  0.78  0.92 | -  0.45-2.54  0.29-2.07  0.36-2.30 | -  0.888  0.612  0.849 |
| **n6: n3** | >5.011  3.944-5.010  3.273-3.943  < 3.272 | Ref  1.23  1.04  0.86 | -  0.81-1.87  0.68-1.60  0.55-1.34 | -  0.335  0.845  0.503 |  | Ref  1.86  0.67  0.70 | -  0.01-4.33  0.23-1.94  0.25-1.93 | -  0.147  0.463  0.489 |
| *Adjusted for maternal age, maternal BMI, Hollingshead socioeconomic status, gestational age, child sex, parity, alcohol use*  *PUFA; Polyunsaturated fatty acids, SGA; Small for gestational age, LGA; Large for gestational age; LA, linoleic acid; AA, arachidonic acid; ALA, alpha-linolenic acid; EPA, eicosapentaenoic acid; DHA, docosahexaenoic acid* | | | | | | | | |

| **Supplementary Table 3: Associations between cord PUFA (mg/ml) and risk of overweight/obesity throughout childhood** | | | | | | | | | | | | |
| --- | --- | --- | --- | --- | --- | --- | --- | --- | --- | --- | --- | --- |
|  |  | **20 month**  **Overweight/Obesity** | | |  | **7 year**  **Overweight/Obesity** | | |  | **13 year**  **Overweight/Obesity** | | |
|  |  | **OR** | **95% CI** | **P-value** |  | **OR** | **95% CI** | **P-value** |  | **OR** | **95% CI** | **P-value** |
| Cord LA | < 0.114 | Ref | - | - |  | Ref | - | - |  | Ref | - | - |
|  | 0.115-0.147 | 1.21 | 0.83-1.76 | 0.312 |  | 1.10 | 0.65-1.84 | 0.740 |  | 1.03 | 0.58-1.83 | 0.911 |
|  | 0.158-0.188 | 1.05 | 0.72-1.54 | 0.786 |  | 1.14 | 0.69-1.87 | 0.550 |  | 1.25 | 0.72-2.20 | 0.431 |
|  | >0.189 | 0.86 | 0.59-1.27 | 0.452 |  | 0.85 | 0.51-1.44 | 0.339 |  | 1.19 | 0.66-2.13 | 0.557 |
|  |  |  |  |  |  |  |  |  |  |  |  |  |
| Cord AA | < 0.144 | Ref | - | - |  | Ref | - | - |  | Ref | - | - |
|  | 0.145-0.189 | 0.85 | 0.58-1.25 | 0.414 |  | 0.76 | 0.46-1.25 | 0.279 |  | 0.80 | 0.46-1.40 | 0.436 |
|  | 0.190-0.265 | 0.94 | 0.65-1.38 | 0.760 |  | 1.01 | 0.61-1.66 | 0.969 |  | 0.98 | 0.56-1.74 | 0.954 |
|  | >0.266 | 0.88 | 0.61-1.29 | 0.524 |  | 0.89 | 0.53-1.50 | 0.668 |  | 0.78 | 0.44-1.36 | 0.379 |
|  |  |  |  |  |  |  |  |  |  |  |  |  |
| Cord Total n-6 | < 0.276 | Ref | - | - |  | Ref | - | - |  | Ref | - | - |
|  | 0.277-0.364 | 0.95 | 0.65-1.38 | 0.773 |  | 0.88 | 0.53-1.44 | 0.606 |  | 0.74 | 0.42-1.29 | 0.285 |
|  | 0.365-0.513 | 0.81 | 0.56-1.18 | 0.277 |  | 0.91 | 0.55-1.50 | 0.712 |  | 0.91 | 0.52-1.60 | 0.750 |
|  | > 0.514 | 0.80 | 0.55-1.16 | 0.235 |  | 0.90 | 0.54-1.51 | 0.692 |  | 0.76 | 0.43-1.35 | 0.349 |
|  |  |  |  |  |  |  |  |  |  |  |  |  |
| Cord DHA | < 0.071 | Ref | - | - |  | Ref | - | - |  | Ref | - | - |
|  | 0.072-0.096 | 1.19 | 0.81-1.73 | 0.372 |  | 0.97 | 0.59-1.58 | 0.891 |  | 1.07 | 0.62-1.84 | 0.803 |
|  | 0.097-0.127 | 1.19 | 0.82-1.74 | 0.356 |  | 1.07 | 0.65-1.77 | 0.790 |  | 0.99 | 0.57-1.70 | 0.965 |
|  | > 0.128 | 0.92 | 0.62-1.35 | 0.661 |  | 0.82 | 0.49-1.37 | 0.445 |  | 1.10 | 0.62-1.95 | 0.751 |
|  |  |  |  |  |  |  |  |  |  |  |  |  |
| Cord n6:n3 | < 2.589 | Ref | - | - |  | Ref | - | - |  | Ref | - | - |
|  | 2.590-3.348 | 0.82 | 0.56-1.20 | 0.313 |  | 1.12 | 0.68-1.85 | 0.652 |  | 0.86 | 0.50-1.50 | 0.687 |
|  | 3.349-5.586 | 0.78 | 0.53-1.15 | 0.210 |  | 1.02 | 0.60-1.70 | 0.956 |  | 0.65 | 0.37-1.16 | 0.182 |
|  | >5.587 | 0.91 | 0.63-1.33 | 0.640 |  | 1.00 | 0.59-1.70 | 0.996 |  | 0.71 | 0.40-1.25 | 0.235 |
| *Adjusted for maternal age, maternal BMI, Hollingshead socioeconomic status, gestational age, child sex, parity, alcohol. Models at 7 and 13 years additionally adjusted for child fish intakes*  *PUFA; polyunsaturated fatty acid, OR, Odds ratio; CI, Confidence intervals; LA, linoleic acid; AA, arachidonic acid; DHA, docosahexaenoic acid* | | | | | | | | | | | | |
